# Proactive Strategies to Prevent Biofilm-Associated Infections: From Mechanistic Insights to Clinical Translation

**DOI:** 10.3390/microorganisms13122726

**Published:** 2025-11-29

**Authors:** María Teresa Hernández-Huerta, Eduardo Pérez-Campos, Laura Pérez-Campos Mayoral, Itzel Patricia Vásquez Martínez, Wendy Reyna González, Efrén Emmanuel Jarquín González, Hanan Aldossary, Ibrahim Alhabib, Lamya Zohair Yamani, Nasreldin Elhadi, Ebtesam Al-Suhaimi, Hector A. Cabrera-Fuentes

**Affiliations:** 1 SECIHTI, Facultad de Medicina y Cirugía, Universidad Autónoma “Benito Juárez” de Oaxaca, Oaxaca 68020, Mexico; mthernandez@secihti.mx; 2 División de Estudios de Posgrado e Investigación, Tecnológico Nacional de México/Instituto Tecnológico de Oaxaca, Oaxaca 68030, Mexico; pcampos@itoaxaca.edu.mx; 3 Centro de Investigación Facultad de Medicina UNAM-UABJO, Facultad de Medicina y Cirugía, Universidad Autónoma “Benito Juárez” de Oaxaca, Oaxaca 68020, Mexico; lperez.cat@uabjo.mx (L.P.-C.M.); vami940508.fmc@uabjo.mx (I.P.V.M.); draqueencitawen@gmail.com (W.R.G.); 4 Dirección General de los Servicios de Salud de Oaxaca, Secretaria de Salud, Servicios de Salud de Oaxaca, Oaxaca 68000, Mexico; drefrenjg@icloud.com; 5 Vice Presidency for Scientific Research and Innovation, Imam Abdulrahman Bin Faisal University, P.O. Box 1982, Dammam 31441, Saudi Arabia; haalhameem@iau.edu.sa (H.A.); ealsuhaimi@iau.edu.sa (E.A.-S.); 6 Department of Clinical Laboratory Sciences, College of Applied Medical Sciences, Imam Abdulrahman Bin Faisal University, P.O. Box 2035, Dammam 31441, Saudi Arabia; ikalhabeeb@iau.edu.sa (I.A.); lzyamani@iau.edu.sa (L.Z.Y.); nmohammed@iau.edu.sa (N.E.); 7 División de Estudios de Posgrado e Investigación, Tecnológico Nacional de México/Instituto Tecnológico de Tijuana, Tijuana 22414, Mexico; 8 R&D Group, Vice Presidency for Scientific Research and Innovation, Imam Abdulrahman bin Faisal University (IAU), P.O. Box 1982, Dammam 31441, Saudi Arabia

**Keywords:** biofilms, antimicrobial resistance, immune evasion, quorum sensing, nanotechnology, biofilm prevention, hormonal modulation

## Abstract

Biofilms are structured microbial communities that adhere to biotic and abiotic surfaces embedded in an autonomous extracellular matrix. These structures contribute to persistent infections, especially in patients with indwelling medical devices, due to their resistance to antimicrobial agents; they have evolved to evade host immune responses. Despite advances in antimicrobial therapies, biofilm-associated infections remain a major challenge in clinical infectious diseases. This perspective explores the underlying mechanisms of biofilm resilience and immune evasion, emphasizing the limitations of conventional treatments and the need to develop pre-emptive measures that focus on preventing biofilm formation rather than implementing a treatment. This work discusses emerging strategies, such as quorum-sensing inhibition, hormonal modulation, matrix-degrading enzymes, anti-adhesive surface modifications, and nanotechnology-based drug delivery, that offer promising avenues to disrupt biofilm formation and maturation. Also offers a shift from the paradigm, looking into proactive prevention rather than treatment, emphasizing clinical translation, scalability, and biocompatibility. Embedding these strategies into routine care could significantly reduce healthcare-associated infections, improve patient outcomes, and mitigate the development of antimicrobial resistance. Our analysis highlights biofilm prevention as a critical frontier in the future of infectious disease management.

## 1. Introduction

**Figure 1:**
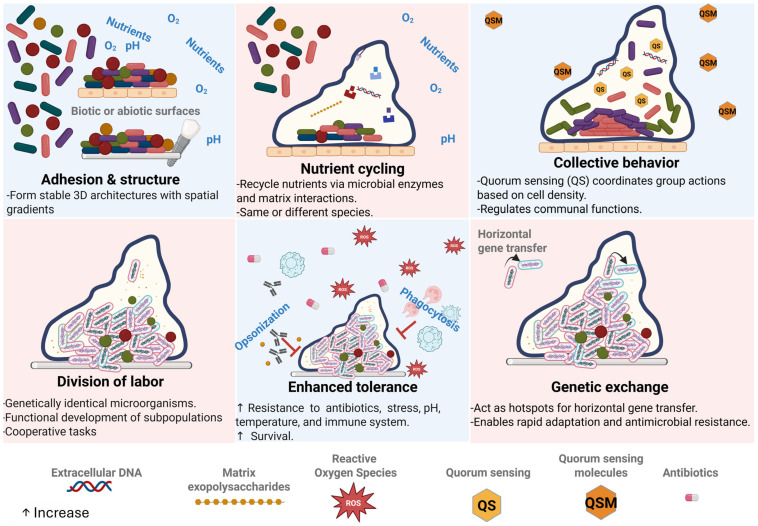
Emergent properties of biofilms include structural stability, antimicrobial resistance, immune evasion, and enhanced horizontal gene transfer.

Biofilms are structured microbial communities encased in an autonomously produced matrix of extracellular polymeric substances (EPS), primarily composed of polysaccharides, proteins, extracellular DNA, and lipids. These components facilitate adhesion to surfaces and confer protection against environmental insults. The specific matrix materials and emergent properties of biofilms result from interactions between resident microorganisms and ecological conditions [1] (Figure 1).

Thus, biofilms protect microorganisms, allowing them to survive in hostile environments [2]. They facilitate the exchange and storage of nutrients, promoting prolonged persistence on surfaces and conferring a significant advantage over free-floating cells [3]. Furthermore, biofilms facilitate the organization of bacteria into subpopulations with specialized functions, increasing collective efficiency [4]. Physical proximity between bacterial cells facilitates the exchange of plasmids and other genetic elements, accelerating adaptation and the spread of antimicrobial resistance [5]. Finally, they allow the creation of diverse microhabitats that benefit the various groups within the biofilm [6]. On the other hand, biofilms fulfill diverse ecological roles by playing a key role in the carbon, nitrogen, and sulfur cycles through processes of degradation, nitrification, denitrification, and sulfate reduction [6]; they form the base of food chains, serving as a fundamental nutrient resource at lower levels of ecosystems [7]; they colonize biological surfaces, for example, in plants they facilitate nitrogen fixation or protect against pathogens; in animals and humans, they allow the formation of beneficial commensal communities or can become pathogenic in the form of chronic infections, such as dental plaque or biofilms associated with medical devices [8]; biofilms have the capacity to modify the environment where they form; and they allow the emergence of new adaptations and the coexistence of species with different lifestyles thanks to the ease of genetic transfer and the chemical heterogeneity in each microbial subpopulation present in the biofilms [2,9].

Quorum sensing (QS), a cell-density-dependent communication system, modulates diverse bacterial phenotypes during biofilm development. It typically involves a two-component regulatory system consisting of a membrane-bound histidine kinase sensor, a cytoplasmic response regulator, and diffusible signaling molecules known as autoinducers, which orchestrate the expression of virulence and biofilm-associated genes. Furthermore, activation of QS pathways promotes biofilm growth and dispersal by regulating essential virulence factors [10].

Biofilms are implicated in the pathogenesis of chronic infections that exhibit marked resistance to conventional antimicrobial therapies, contributing to substantial global morbidity and mortality, particularly in patients with indwelling medical devices and/or compromised immune systems [11,12].

Biofilm-associated resistance arises from multiple factors, including reduced bacterial metabolic activity, restricted antibiotic penetration due to the EPS matrix, horizontal gene transfer within the community, and the presence of phenotypically distinct persister cells that exhibit transient tolerance to antimicrobials [13]. Antibiotic therapy often fails to eradicate infections caused by biofilms, which are responsible for approximately 65% of all human infections [14]. Therefore, biofilms represent a complex clinical problem and a growing threat to public health.

## 2. The Clinical Challenge of Biofilm

Biofilm formation is a complex process and is triggered by the adhesion of free-floating (planktonic) microorganisms to surfaces [15]. The first step involves the reversible adhesion of microorganisms to biotic or abiotic surfaces through weak acid-base, hydrophobic, van der Waals, and electrostatic interactions [16]; here, these microorganisms are polarly bound. Subsequently, the microorganisms lie flat on the surfaces and seek irreversible attachment, which is responsible for the development of resistance to physical factors that hinder biofilm formation [3,5]. Subsequently, colonization and proliferation occur.

Adhered microorganisms form microcolonies through multiplication and aggregate formation within the EPS, all in high concentrations of cyclic (3′-5′)-dimeric guanosine monophosphate (c-di-GMP) and through stronger interactions between adhesive proteins that bind collagen, lipopolysaccharides, flagella, and pili. Furthermore, significant amounts of EPS are produced [3,5,17] and continuous biofilm maturation facilitates microbial attachment to surfaces, thereby stabilizing the biofilm’s 3D structure [3]. The adhesive proteins present in microbial biofilms vary according to the species, some examples are pili in *Pseudomonas aeruginosa*, *Neisseria*, and *Vibrio*, which allow mannose recognition, adhesion, and motility [18,19]; microbial surface components recognizing adhesive matrix molecules (MSCRAMMs), surface adhesins that bind to surfaces, extracellular matrix, or host cells of Gram-positive bacteria [20]; and self-transporting adhesins that promote self-aggregation secreted by Gram-negative bacteria [21,22]. Biofilm maturation is a necessary step that promotes cell aggregation, forming potent niches that are highly resistant to processes that support evasion of host immune responses, oxidative damage, and metal cation damage. These cell aggregates are also resistant to antimicrobial agents, leading to continued survival.

Further modifications to the microenvironment can induce bacteria to leave the biofilm matrix, ultimately disseminating to new anatomical locations and spreading the infection [23]. Biofilm formation plays a crucial role in the development of antibiotic resistance. It contributes to the formation of persister cells, which are responsible for the resilient, often unmanageable persistence of microbial infections [24,25].

Implanted medical devices that are frequently colonized by biofilms include urinary catheters, pacemakers, mechanical heart valves, vascular grafts, and endotracheal tubes [26,27]. For example, orthopedic devices are commonly infected with *Staphylococci* and Gram-negative *bacilli*, while *Pseudomonas aeruginosa* and *Staphylococci* frequently colonize endotracheal tubes and contact lenses. Intravascular catheters tend to harbor *Staphylococci*, *Enterococci*, and Gram-negative *bacilli*, while pacemakers and heart valves are commonly infected with *Staphylococci* and *Streptococci*. Other devices, such as respiratory equipment, urinary catheters, cerebrospinal shunts, and dental implants, are associated with pathogens such as *Acinetobacter baumannii*, *Enterococcus faecalis*, *Staphylococcus aureus*, *Propionibacterium*, *Prevotella intermedia*, and *Actinobacillus*. The development of biofilms in medical devices depends on several factors. These devices can be made of polymers, metals, or ceramics, and each material affects the likelihood of biofilm infection. Hydrophobic polymers such as silicone, polyvinyl chloride, and polytetrafluoroethylene facilitate bacterial adhesion. In contrast, metals like titanium and stainless steel, as well as hydrophilic ceramics such as zirconia and hydroxyapatite, tend to decrease initial colonization. Furthermore, surface properties determine the risk of infection: (a) hydrophobic, rough, or positively/neutrally charged surfaces promote adhesion, and (b) hydrophilic surfaces limit initial contact. The chemical composition and specific coatings also influence the adhesion of certain pathogens [28]. The risk increases when devices are worn for extended periods, exposed to bodily fluids, or placed in environments with constant flow, as this promotes colonization by microorganisms such as *Staphylococcus epidermidis*, *Staphylococcus aureus*, and *Pseudomonas aeruginosa*. This diversity of characteristics underscores the clinical importance of biofilm formation across a wide range of medical devices [29]. Infections associated with biofilms include periodontitis, osteomyelitis, pulmonary infections in cystic fibrosis [30], endocarditis [31], dental plaque, chronic tonsillitis, and chronic laryngitis [32,33]. In addition, patient-related factors increase the risk of device-related infections. Among the most relevant are immunosuppression, diabetes, smoking, and diseases such as kidney failure or the need for hemodialysis. Finally, many of these infections originate from contamination of the device during insertion, either by microorganisms from healthcare personnel or the patient themselves, which facilitates biofilm formation [34].

Understanding the mechanisms involved allows us to develop effective therapeutic targets and anti-biofilm strategies, particularly through surface modifications, as these represent critical points for therapeutic intervention.

Therefore, biofilm prevention represents a more effective and viable strategy for treatment, both in terms of its effectiveness and long-term benefits, as well as its cost and patient quality of life. Once established, due to their nature, biofilms are extremely difficult to eradicate, leading to persistent infections, perioperative complications, and longer hospitalizations, which result in rising annual costs for hospitals and governments.

Furthermore, it is essential to consider that treating a biofilm-induced infection often requires high doses of antibiotics for prolonged periods, with limited success and risk of altering the patient’s microbiome and promoting antimicrobial resistance [35]. In many cases, treatment involves the surgical removal of contaminated medical devices, which carries a high risk to the patient’s health and additional expenses for healthcare systems. In contrast, prevention strategies are more effective, less costly, and sustainable over time. Preventing biofilm formation from its initial adhesion stage prevents the infection from progressing to a chronic, therapy-resistant state.

Thus, investing in prevention reduces the risk of clinical complications and the associated economic burden, combats antimicrobial resistance, protects vulnerable populations, and strengthens the quality of care in modern healthcare systems.

## 3. Immune Response to Biofilms

The host immune response is activated when virulence factors from certain microorganisms are detected, initiating host cell interactions that stimulate the release of substances that promote infection. However, pathogenic microorganisms develop mechanisms to evade the immune response, survive, and spread within the host. Furthermore, several studies have demonstrated that virulence factors promote biofilm formation and contribute to complications associated with antimicrobial resistance and the survival of pathogens on abiotic surfaces [36].

Biofilm-embedded bacteria display enhanced resilience to environmental and host-derived stressors compared to their planktonic counterparts, including desiccation, oxidative stress, UV radiation, exposure to innate and adaptive immune effectors, and antimicrobial agents. The host immune system lacks a dedicated response against biofilms; instead, biofilms employ multifaceted evasion strategies, including immune modulation, secretion of anti-inflammatory mediators, and physical shielding that impairs phagocytosis and complement activation. For example, biofilms can act as physical barriers that help bacteria avoid detection and phagocytosis. Additionally, they can activate genetic response regulators, switches, or suppressors that influence the activity of immune cells [37].

Planktonic infections, which occur without a biofilm, typically trigger a rapid response from neutrophils (PMNs) and result in the pro-inflammatory activation of macrophages. M1 macrophages promote Th1 polarization of CD4 cells by facilitating complement-mediated phagocytosis of intracellular pathogens and type I inflammation [38]. These macrophages play a crucial role in eliminating the infection due to their strong bactericidal activity. Furthermore, Th1 and Th17 cells are often recruited to enhance phagocyte activation and establish memory populations that enable a swift response in case of reinfection. In biofilm-associated infections, macrophages often adopt an M2-like phenotype, promoting tissue remodeling and immune tolerance rather than effective bacterial clearance, thereby contributing to the persistence of chronic infections. Although PMNs are still present in these cases, they are generally ineffective at clearing the infection [39].

## 4. Hormonal Modulation of Biofilm Formation: Endocrine, Metabolic, and Immune Interplay

### 4.1. Endocrine Regulation: Hormones as Biofilm Modulators

The concept of microbial endocrinology illustrates the intricate inter-kingdom crosstalk between host endocrine signals and bacterial QS systems, notably in *Pseudomonas aeruginosa*. Several human hormones, including insulin, sex steroids, and stress-related hormones, have been shown to influence bacterial virulence by interacting with bacterial QS pathways [40]. These hormonal signals can either suppress or enhance biofilm formation, suggesting a hormone-specific and pathogen-specific regulatory mechanism. For example, insulin has been implicated as a QS mimic that can modulate the expression of bacterial virulence factors, including those responsible for biofilm development [41]. While in some models, insulin suppresses biofilm growth, in diabetic contexts, insulin was found to enhance *Pseudomonas aeruginosa* biofilms by upregulating intracellular cyclic-di-GMP levels, a key regulator of bacterial adhesion and matrix production [42]. Notably, sex hormones such as estrogen and testosterone have also been proposed to affect bacterial colonization patterns, possibly contributing to sex differences in infection severity [19].

### 4.2. Metabolic Context: Biofilms at the Crossroads of Glucose, Insulin, and Metabolism

Host metabolic status plays a crucial role in shaping biofilm development. In individuals with diabetes, elevated serum glucose and insulin levels alter the microenvironment, promoting bacterial persistence. Multiple studies have demonstrated that insulin enhances the biofilm-forming capabilities of pathogens such as *Escherichia coli* and *Pseudomonas aeruginosa*, particularly under high-glucose conditions, possibly through interactions with bacterial receptors or signaling pathways [43,44].

Further complicating this relationship is the role of immunometabolic dysfunction in diseases such as metabolic syndrome and obesity, which impair cellular energy metabolism and exacerbate inflammatory responses. Metabolic disorders downregulate key immunometabolic pathways, including the PI3K-AKT-mTOR and TLR signaling pathways, thereby weakening the host’s ability to respond to biofilm-associated pathogens [45,46]. Moreover, adipokines such as resistin and pro-inflammatory cytokines from adipose tissue contribute to chronic inflammation and impaired healing, creating favorable niches for biofilm persistence in both periodontal and systemic infections [47,48].

### 4.3. Immune Interface: Hormonal Modulation of Host–Biofilm Immune Dynamics

Biofilms are adept at evading immune responses through the suppression of immune cell activation, the secretion of anti-inflammatory mediators, and the shielding provided by extracellular polymeric substances. Hormones can indirectly influence these dynamics by modulating immune cell recruitment and function. For instance, insulin treatment in diabetic mice increased neutrophil infiltration and lysis, leading to the release of extracellular DNA that enhanced biofilm matrix development and antimicrobial resistance [49]. Concomitantly, insulin suppressed macrophage activity, reducing the clearance of biofilm components and exacerbating chronic infection. Additionally, altered cytokine profiles—such as increased IL-4 and earlier IFN-γ expression—were reported in insulin-treated diabetic mice, further contributing to a skewed and ineffective immune response against biofilm pathogens [42]. These findings suggest that the insulin-glucose axis not only directly affects bacterial behavior but also reprograms the immune landscape, often favoring biofilm persistence. On a therapeutic note, immune-enhancing compounds such as vitamin C have shown promise in restoring immune clearance of biofilms by downregulating the recA gene, mitigating SOS response induction, and restoring antibiotic sensitivity in multidrug-resistant *Escherichia coli* [50]. Furthermore, obesity promotes the secretion of hormones such as leptin, insulin, and free fatty acids, as well as inflammatory molecules (RAGE, LRP1, cytokines, particularly IL-6 and TNF-α) [51]. These findings highlight a promising intersection of immunometabolism and biofilm control.

As summarized by Wu et al. (2025) [52], recent advances in microbial endocrinology reveal that many human hormones directly influence microbial growth, virulence, and biofilm behavior through inter-kingdom signaling. Host hormones such as catecholamines (epinephrine, norepinephrine, dopamine), opioid peptides (dynorphin, β-endorphin), natriuretic peptides, gastrin, serotonin, insulin, and sex hormones (estrogen) have all been shown to modulate bacterial quorum-sensing systems and virulence gene expression. These interactions can enhance biofilm formation, motility, and persistence in pathogens, including *Pseudomonas aeruginosa*, *Escherichia coli*, *Vibrio* spp., and *Helicobacter pylori*. Such hormone-mediated crosstalk suggests that endocrine disturbances, such as stress, diabetes, or metabolic disorders, may inadvertently favor biofilm-associated infections. Despite growing evidence, the specific microbial hormone receptors and signaling pathways remain only partially defined and represent an important target for future anti-biofilm strategies.

### 4.4. Targeted Strategy Against Biofilms Using Hormonal and Metabolic Controllers

An innovative strategy to control biofilms involves modulating host metabolic and hormonal pathways that indirectly influence microbial behavior, immune responses, and the structural integrity of biofilm communities. Based on the above references, biofilms are known to exploit host hormones, such as leptin and insulin, as well as metabolic cues, to enhance quorum sensing, virulence expression, and immune evasion.

Additionally, the gut microbiota, which coevolved with the host, supports immune development, nutrient absorption, and protection against pathogenic colonization. It suppresses pathogens through metabolic competition, niche occupation, and immune activation, while pathogens evolve to overcome this defense. Decoding this dynamic interplay offers promising avenues for infection control and immune-modulating therapies [53].

Leptin, a hormone primarily associated with satiety and energy regulation, has been shown to play critical roles in immune modulation and neuro-immune signaling [30]. Its dysregulation, commonly observed in obesity, alters cytokine profiles and immune cell responsiveness, potentially creating an immunosuppressive microenvironment that favors pathogens’ persistence. Thus, leveraging leptin-modulating agents to restore metabolic balance may indirectly suppress associated pathogens. In addition to host-derived hormones, microbial metabolites, such as short-chain fatty acids (SCFAs), play key roles in modulating immune and metabolic signaling pathways relevant to biofilm control. SCFAs—including acetate, propionate, and butyrate—serve as signaling molecules that influence G-protein-coupled receptor activity, histone deacetylase inhibition, and immune cell metabolism, thereby linking microbial metabolic output with host immunoregulation. Recent studies highlight *Lactobacillus* spp. as potential SCFAs producers that, although not traditionally associated with biofilm regulation, can modulate microbial community dynamics and host inflammatory responses. Advances in metabolic engineering and synthetic biology to optimize SCFAs yield from probiotic *Lactobacillus* strains may therefore represent a novel adjunctive strategy for managing inflammatory and biofilm-associated disorders [54].

## 5. Emerging Biofilm Prevention Strategies

Bacterial biofilm-associated infections are caused by groups of microorganisms originating from multiple species. This multispecies community includes bacteria, fungi, and occasionally components derived from the host, forming intricate and resilient microbial communities [55,56]. As a consequence of biofilm formation, they exhibit distinct characteristics within the multispecies interactions, such as metabolic activity, horizontal gene transfer, QS communication, antimicrobial tolerance, immune responses, and alterations in spatial organization and structure [57,58]. A recent study reported that increases in antimicrobial resistance and pathogenicity are driven by multispecies biofilms. However, fungal biofilms can increase bacterial drug resistance as demonstrated by synergistic interactions between *Candida albicans* and *Pseudomonas aeruginosa* or *Staphylococcus aureus* [59].

The extracellular matrix of biofilms forms a diffusion barrier that impairs the penetration of antibiotics and biocidal agents, effectively enhancing microbial survival and promoting phenotypic tolerance [60]. A biofilm helps pathogenic bacteria evade phagocytosis and resist attacks from antimicrobial substances, including reactive oxygen and nitrogen species. This ability to withstand immune responses and antibiotic treatments makes infections caused by bacteria residing in biofilms particularly challenging to treat [61].

Preventing biofilm formation is easier than eradicating it. The most common strategies for preventing biofilm formation include surface modification and coating, delivery systems, and anti-biofilm approaches. We can use several methods to combat bacterial infections due to their biofilm formation (Table 1).

These strategies include the use of bacterial adhesin inhibitors, surfactants, and dispersing enzymes, which prevent early biofilm formation and limit initial colonization on medical surfaces or tissues. Other potential approaches include quorum-sensing inhibitors (QSIs) and matrix-degrading enzymes, such as DNases and dispersin B, which can break down biofilm structures. These tools destabilize the mature biofilm structure, making it more susceptible to antimicrobial treatments. Additionally, we can use enzymes that inhibit EPS synthesis. Combining antibiotics with matrix-disrupting enzymes and using drugs that enhance penetration and target persister cells (which contribute to antibiotic tolerance) are also promising, somewhat personalized strategies. Inducing controlled dispersal, for example, with nitric oxide, allows us to target newly dispersed, more vulnerable bacterial cells. Furthermore, blocking dispersal signals can help prevent reinfection. Together, these methods can improve our ability to enhance therapeutic efficacy and reduce overreliance on antibiotics, which could help mitigate the global problem of antimicrobial resistance.

A thorough understanding of the molecular and environmental determinants is required for developing effective preventive therapies [38]. The role of these ecological interactions in determining treatment failure and infection persistence is becoming increasingly acknowledged. Preventive measures must therefore take interspecies communication and microbial dynamics into consideration. Targeting metabolic cross-feeding networks that promote polymicrobial survival, enzymatic biofilm matrix breakdown, and QS inhibition are examples of emerging strategies [62]. By altering microbial community structure, microbiome-based therapies, such as probiotics, modified commensal strains, and prebiotics, have shown promising results in reducing the formation of pathogenic biofilms [63]. The prevention and treatment of biofilm-associated infections may be significantly enhanced by combining these microbiota-aware approaches, strategies, and interventions with conventional infection control and biomaterial surface modifications. Future studies should focus on host reactions, in vivo interactions, and the potential of natural materials and polymeric devices to optimize treatment outcomes while mitigating the clinical burden of infections and reducing the burden of diseases linked to multispecies biofilms [38,42].

Beyond endocrine signaling, bacterial communication through QS networks also contributes to biofilm formation and host interaction. In *Pseudomonas aeruginosa*, N-acyl-homoserine-lactone-dependent QS systems regulate virulence, metabolism, and biofilm maturation while simultaneously modulating host calcium signaling, mitochondrial dynamics, and cytoskeletal responses. These interconnected pathways illustrate the bidirectional nature of bacteria–host communication and suggest that disrupting such signaling could attenuate infection severity and antibiotic resistance [64]. Host immunometabolism also shapes bacterial persistence and biofilm adaptation. Upon infection, macrophages undergo metabolic reprogramming that fuels inflammatory and bactericidal responses; however, pathogens such as *Pseudomonas aeruginosa*, *Staphylococcus aureus*, and *Salmonella* can exploit overlapping metabolic pathways to survive within inflamed tissues. By aligning their own metabolic networks with those of activated host cells, these bacteria enhance antibiotic tolerance, promote biofilm maturation, and generate persister cells that sustain chronic infection. This shared metabolic circuitry between host and pathogen underscores the immunometabolic regulation of biofilm resilience and highlights the potential of targeting host–pathogen metabolism to reduce infection persistence [65]. Biofilm formation relies on sophisticated cell-to-cell communication systems regulated by small signaling molecules, including N-acyl homoserine lactones (AHLs), autoinducer-2 (AI-2), and autoinducing peptides (AIPs). These QS pathways control gene expression related to adhesion, maturation, virulence, and antibiotic resistance. Targeting these systems through enzymatic degradation or signal inhibition (quorum quenching) represents a promising antibiofilm approach that can complement conventional antimicrobial therapies [66].

**Table 1:** Emerging Biofilm Prevention Strategies.

Emerging Strategy	Mechanism of Action	Advantages	Limitations	Clinical/Practical Applications	Examples/Notes	Ref.
SACs	Release of antimicrobials or reactive agents in response to microbial signals	High specificity; activity triggered only when bacteria are present	High production cost; potential cytotoxicity	Catheters, endotracheal tubes, orthopedic implants, wound dressings	Light-activated coatings, antibiotic-eluting polymers	[67,68]
MDEs	Enzymatic degradation of EPS components (DNA, proteins, PS)	Enhances antibiotic penetration and disrupts mature biofilms	Limited stability; immunogenicity concerns	Wound care, infected implants, chronic ulcers	DNase I, dispersin B, proteinase K, subtilisin	[69,70,71,72]
QSIs	Block communication signals required for adhesion, maturation, and virulence	Low likelihood of resistance; targets early biofilm formation	Limited commercial availability; variable efficacy	Prevention of device-associated infections; coatings; topical applications	Synthetic furanones, ajoene, and plant-derived inhibitors	[73,74,75]
Functionalized NPs	Disrupt bacterial membranes; generate ROS; penetrate biofilm matrix	Potent against MDR organisms; broad-spectrum	Potential toxicity; environmental accumulation	Antimicrobial catheters, dental materials, and topical antimicrobials	Ag-S-1, AgNPs + Ca(OH)_2_	[76,77,78,79,80]
Engineered Bacteriophages	Target and lyse specific biofilm-forming bacteria; self-amplifying	High specificity; active in dense biofilms	Regulatory challenges; narrow host range	Chronic device-related infections; wound therapy	Phage cocktails, engineered biofilm-penetrating phages	[81,82]
AMPs	Disrupt bacterial membranes; inhibit adhesion	Low resistance potential; broad-spectrum	Degradation in physiological environments	Coatings for implants, topical gels, and catheter lock solutions	Melittin, LL-37, indolicidin	[83,84,85]
Anti-Adhesive Surface Modification	Alters physicochemical properties to prevent bacterial attachment	Prevents early colonization without antibiotics	Ineffective once biofilm forms	Titanium implants, dental implants, vascular grafts	PEGylation, zwitterionic coatings, plasma-treated surfaces	[86,87,88]
Probiotics and Competitive Exclusion	Occupy niches and inhibit pathogens through competition and metabolite secretion	Safe, biocompatible; modulates microbiome	Requires sustained application; strain-dependent efficacy	Urogenital/gastrointestinal mucosa, oral cavity	*Lactobacillus* spp., *Bacillus subtilis*	[89,90,91]

SACs: Smart Antimicrobial Coatings; EPS: extracellular polymeric substances; DNA: Deoxyribonucleic Acid; PS: polysaccharides; DNase I: Deoxyribonuclease I; MDEs: Matrix-Degrading Enzymes; QSIs: Quorum Sensing Interference or Quorum Sensing Inhibitors; NPs: Nanoparticles; ROS: Reactive Oxygen Species; MDR: Multidrug-resistant; Ag-S-1: Ag-silicalite-1; AgNPs: Silver Nanoparticles; Ca(OH)_2_: calcium hydroxide; AMPs: Antimicrobial peptides; PEG: Polyethylene glycol.

Biomedical device companies have also incorporated a wide variety of anti-adhesive and anti-biofilm coatings designed to prevent microbial colonization and reduce the risk of implant-associated infections [25]. Among the most common strategies are photothermally activated antibacterial surfaces, coatings containing phenolic acids (such as tannic acid), and metal complexes that inhibit the adhesion of *Escherichia coli* and *Staphylococcus aureus*. Gold, silver, or zinc nanoparticles are also used, as well as bioactive materials such as hydroxyapatite, chitosan, or black phosphorus, which provide additional antimicrobial or anti-adhesive properties. Many devices use hydrophilic polymers, zwitterionic copolymers, or positively charged coatings to reduce initial interactions between the surface and bacteria. Others use more advanced systems, such as encapsulated photosensitizing agents, nitric oxide releasers, or coatings that release antibiotics (ciprofloxacin, diclofenac, or vancomycin) in a controlled manner. These technologies are applied to a wide range of devices—from catheters, stents, and contact lenses to dental, orthopedic, and subcutaneous implants—providing an effective barrier against pathogens such as *Pseudomonas aeruginosa*, *Staphylococcus aureus*, *Streptococcus* spp., and *Bacillus subtilis*.

Preventing microbial adhesion through engineered surface modification is one of the most promising strategies to reduce device-related infections. A growing body of evidence demonstrates that modifying surface chemistry or topography can significantly limit early microbial attachment and, consequently, biofilm initiation. As highlighted in a comprehensive review on anti-adhesive medical device technologies, two major approaches exist: (1) incorporating biocidal agents into the device surface, either through release-based or contact-active mechanisms, and (2) altering the physicochemical properties of the surface to prevent adhesion altogether [86]. While biocide-releasing coatings can be effective, their clinical application remains controversial due to concerns regarding toxicity and the selection of resistant strains. Anti-adhesive strategies, by contrast, focus on manipulating hydrophobicity, charge, roughness, and nanoscale structuring to minimize non-specific microbial attachment. Such approaches aim to reduce adhesion without exerting selective antimicrobial pressure, making them an attractive avenue for safer, long-term prevention. However, the literature also emphasizes that clinical evidence demonstrating improved outcomes with these modified surfaces is still limited, and further research is required to identify the most effective surface parameters for reducing microbial burden [86]. Recent advances demonstrate the utility of polymer surface modification in overcoming inherent material limitations. For example, polyetheretherketone (PEEK), although widely used in orthopedic and dental applications, exhibits strong hydrophobicity and poor intrinsic surface adhesion properties. Plasma treatment can transiently enhance surface hydrophilicity; however, these effects typically diminish rapidly. A combined plasma and PEG-silane strategy was shown to achieve stable long-term hydrophilic modification, as indicated by sustained decreases in contact angle and long-term retention of functional groups such as hydroxyl, peroxide, and carboxylic acids. These modifications improve surface energy and adhesion strength for prolonged periods—up to 48 days—far exceeding plasma treatment alone [87]. This evidence highlights how hybrid chemical treatments can provide durable anti-adhesive and biofilm-resistant properties to polymeric biomaterials. In addition to polymer modification, metallic materials—particularly titanium and its alloys—have been the focus of extensive surface engineering research due to their widespread use in orthopedic and dental implants. Although titanium exhibits excellent biocompatibility and mechanical performance, it still requires surface modification to enhance osseointegration and reduce bacterial colonization. Advanced techniques such as plasma spraying, physical vapor deposition, sol–gel coatings, and micro-arc oxidation have been shown not only to improve surface microstructure and mechanical stability but also to increase antibacterial performance. These modifications create micro-/nano-structured surfaces that support tissue integration while minimizing microbial attachment. Emerging composite coating strategies further combine multiple technologies to achieve synergistic improvements in bioactivity, mechanical strength, and antimicrobial effects [88]. Future research is expected to integrate multifunctional coatings that simultaneously optimize mechanical, biological, and antimicrobial characteristics for next-generation implants.

**Figure 2:**
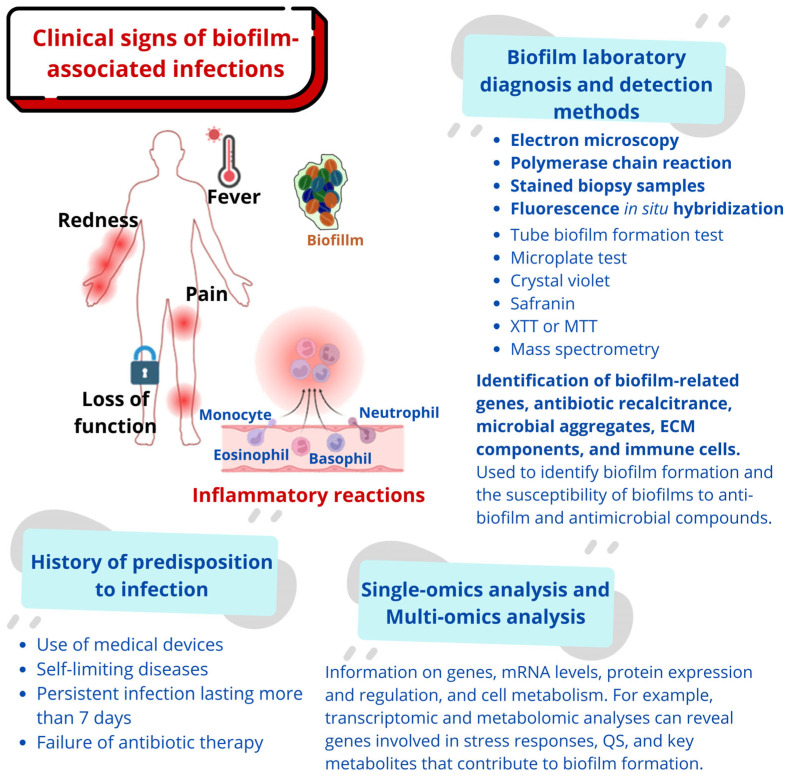
Biofilm detection and diagnosis methods. ECM: extracellular matrix; XTT: 2,3-bis (2-methoxy-4-nitro-5-sulfophenyl)-5-[(phenylamino) carbonyl 2H-tetrazolium hydroxide; MTT: 3-(4,5-dimethylthiazol-2-yl)-2,5-diphenyltetrazolium bromide; QS: quorum sensing.

Collectively, these findings underscore the importance of non-biocidal, surface-based prevention strategies as an essential component of modern anti-biofilm design. Integrating chemical, physical, and hybrid surface treatments offers a promising approach to reducing device-associated infections while avoiding the drawbacks of antimicrobial release systems. It is also vital to consider biofilm detection and diagnosis methods, which are essential for prevention and guidance of clinical treatment [92,93], as shown in Figure 2.

Collectively, these solutions represent a multifunctional approach that combines physical, chemical, and pharmacological properties to prevent biofilm formation and ensure the safe and long-term functioning of implantable medical devices.

## 6. Limitations

While numerous anti-biofilm strategies show promising results in preclinical models, their clinical translation remains limited by several factors. Another critical limitation is the incomplete understanding of microbial gene expression regulation in drug-resistant biofilm communities. Biofilm-embedded bacteria display differential transcriptional profiles compared with planktonic cells, leading to the up-regulation of quorum-sensing genes, efflux pumps, and stress-response regulators that collectively enhance antimicrobial tolerance. Crosstalk between host-derived hormones and bacterial QS systems further modulates these transcriptional programs, influencing virulence and persistence [31,43]. In addition, immunometabolic adaptation in inflamed tissues enables pathogens such as *Pseudomonas aeruginosa* and *Staphylococcus aureus* to reprogram their metabolic and gene-expression networks toward persistence and antibiotic tolerance [44]. The dynamic regulation of these genes remains poorly characterized, particularly under host-like microenvironments, underscoring the need for multi-omics and transcriptomic approaches to unravel the regulatory circuits controlling biofilm resilience. Moreover, microbial metabolites such as SCFAs may indirectly influence global transcriptional responses, linking metabolic signaling to gene regulation in mixed-species biofilms [33].

Additionally, consider their ability to alter the complexity of in vivo biofilm environments, variability in host responses, and the challenges of delivering agents effectively at infection sites. Safety, scalability, and regulatory hurdles, especially for novel therapeutics such as engineered bacteriophages or functionalized nanoparticles, must be thoroughly addressed. Furthermore, the lack of standardized biofilm models for clinical testing hinders comparative assessment. Bridging these gaps will require interdisciplinary collaboration and sustained investment in translational research. Future efforts should focus on refining delivery systems for precision targeting of biofilms, developing immunomodulatory therapies to overcome tolerance mechanisms, and validating anti-biofilm agents in rigorous clinical trials. Emphasis should also be placed on scalable, biocompatible technologies that can be integrated into routine care settings, particularly in high-risk environments such as Intensive Care Units and surgical wards. Establishing global standards for anti-biofilm efficacy testing and encouraging collaborative innovation across microbiology, materials science, and clinical medicine will be essential to accelerate progress in this field.

## 7. Conclusions

Biofilm-related infections pose a significant challenge in clinical practice due to their complex structure, resistance to antimicrobial agents, and ability to evade the host immune response. As a result, prevention is not only more cost-effective but also more sustainable and clinically beneficial. To improve clinical outcomes, it is essential to focus on early adhesion prevention, disrupt biofilm maturation, and combine antimicrobial and immune therapies. Future efforts should prioritize the long-term efficacy, safety, detection methods, and scalability of these strategies in real-world settings. Ultimately, promoting biofilm prevention as a fundamental aspect of infection control will enhance patient safety, reduce antimicrobial resistance, and improve the quality and sustainability of modern healthcare.

## Data Availability

No new data were created or analyzed in this study. Data sharing is not applicable to this article.

## Abbreviations

The following abbreviations are used in this manuscript:

AgNPs	Silver Nanoparticles
AMPs	Calcium Hydroxide
c-di-GMP	Cyclic Dimeric Guanosine Monophosphate
EPS	Extracellular Polymeric Substances
MTT	3-(4,5-dimethylthiazol-2-yl)-2,5-diphenyltetrazolium bromide
PEEK	Polyetheretherketone
PEG	Polyethylene Glycol
QS	Quorum Sensing
QSIs	Quorum Sensing Inhibitors/Quorum Sensing Interference
SCFAs	Short-chain fatty acids
TLR	Toll-Like Receptor
XTT	2,3-bis(2-methoxy-4-nitro-5-sulfophenyl)-5-[(phenylamino)carbonyl]-2H-tetrazolium hydroxide
